# Job satisfaction among Swedish police officers: The role of work-related stress, gender-based and sexual harassment

**DOI:** 10.3389/fpubh.2022.889671

**Published:** 2022-07-18

**Authors:** Arian Rostami, Mehdi Ghazinour, Monica Burman, Jonas Hansson

**Affiliations:** ^1^Department of Epidemiology and Global Health, Faculty of Medicine, Umeå University, Umeå, Sweden; ^2^Umeå Centre for Gender Studies, Faculty of Social Sciences, Umeå University, Umeå, Sweden; ^3^Police Education Unit, Faculty of Social Sciences, Umeå University, Umeå, Sweden

**Keywords:** job satisfaction, work stress, gender-based harassment, sexual harassment, gender, police officers

## Abstract

The aim of this cross-sectional study is to increase our understanding of job satisfaction in Swedish police officers by taking into account work-related stress, and sexual and gender-based harassment. Data were collected from 152 police officers working in vulnerable areas in Stockholm using sociodemographic questions, the Police Stress Identification Questionnaire (PSIQ), Sexual and gender-based harassment questions, and Job Descriptive Index (JDI). The obtained results indicated that male and female police officers reported the highest satisfaction in “people on your present job.” The lowest score of job satisfaction in both male and female police officers was related to “opportunity for promotion” and then “pay.” There were no significant differences in the subscales of job satisfaction between male and female police officers. The older and more experienced officers, the less satisfaction was reported in “job in general” and more satisfaction reported in “pay.” Comparing job satisfaction between patrol officers and those officers who worked in internal services showed police patrol officers had higher job satisfaction in “job in general,” “work in the present job,” “opportunity for promotion” and “supervision” compared to their counterparts in internal services. There were not any significant differences between the subscales of job satisfaction between male and female police officers. There was not any significant association between job satisfaction subscales and having experience of sexual or gender-based harassment. Among various subscales of police stressors, organizational stress was in negative relation with three domains of job satisfaction; “job in general,” “pay” and “supervision.” Also, hierarchical multiple regression analyses showed organizational stress was most often of predictive impact related to various job satisfaction domains in police officers.

## Introduction

Job satisfaction is a fundamental concern to both the employee and the overall organization. Job satisfaction is associated with reduced burnout and turnover, and it increases employees' commitment, motivation, and intention to continue working in the organization (1, 2). Research shows a meaningful relationship between job satisfaction and employees' mental and psychological problems such as burnout, low self-esteem, depression, and anxiety (3, 4). Also, as Whitman et al. (5) explained in a meta-analysis study, job satisfaction is correlated with higher performance, productivity, customer satisfaction, and organizational citizenship behaviors. Therefore, job satisfaction can influence organizational efficacy, performance, and quality of services on the societal level (6, 7).

Job satisfaction is associated with perceived work-related stress, and work stress is considered to be a predictor of job satisfaction (8–10). Research confirms that police work is a stressful occupation, and cumulative everyday job stress and traumatic incidents that police officers deal with can increase the risk of negative consequences such as job burnout, health issues, low job satisfaction, and poor performance (11–13). In addition, female police officers face extra work-related stress because the police organization is a male-dominant system, with values and expectations rooted in masculine norms (14, 15). Women have reported some of their work challenges in police organization such as promotion and benefits inequality, underestimation of their abilities at work, insufficient support, and sexual and gender-based harassment by colleagues and citizens (16, 17). Furthermore, gender-based and sexual harassment are forms of gender discrimination that violate human rights and are a crucial source of work-related stress among police officers, and this needs more attention in police research.

Although research on police work has been developed in Sweden during the last decade (after police education was integrated into the academic setting), systematic evidence-based policing still needs to be improved. There is a lack of study on job satisfaction in police officers in Sweden and in relation to other risk factors such as work-related stress and gender-based and sexual harassment. In the current study, we aimed to increase our understanding of job satisfaction among Swedish police officers by taking into account work-related stress, gender, and sexual and gender-based harassment. The study sought answers to the following questions: (a) Are there gender differences in different dimensions of job satisfaction among police officers?; (b) How are sociodemographic variables, work-related stress, and sexual and gender-based harassment associated with job satisfaction in police officers?; and (c) Which sociodemographic variables and which domains of work-related stress and sexual/gender-based harassment can explain variations in job satisfaction in police officers?

### Job satisfaction

There are different approaches toward job satisfaction as a concept. Locke (18) defined job satisfaction and dissatisfaction as emotional states resulting from the perceived relationship between one's expectations from one's job and the appraisal of different aspects of the job. Hopkins (19) defined job satisfaction as the fulfillment of the needs related to one's job. According to Hoppock (20), job satisfaction comprises psychological, physiological, and environmental factors that make employees feel satisfaction regarding their job. Although job satisfaction can be affected by different factors, this multifaceted concept refers to an internal and subjective state that is associated with a feeling of fulfillment with one's job (21). Herzberg (22) in his motivation-hygiene theory differentiated between the factors causing job satisfaction and dissatisfaction. Job satisfaction results from motivation factors such as achievement, recognition, responsibility, and promotion, while the hygiene factors such as organization policy and administrative practices, supervision, interpersonal relations, environmental working conditions, job security, and salary can lead to job dissatisfaction. Spector (23) defined job satisfaction as a multi-dimensional concept of employees' feelings related to both the work itself (intrinsic factors) and the working environment (extrinsic factors). Smith et al. (24) explained that job satisfaction comprises some facets such as the job itself, promotion, pay, supervision, and colleagues and that general job satisfaction can be affected by all of these mentioned facets. According to their explanation, job satisfaction encompasses the individuals' appraisals and feelings related to these facets.

### Gender and other demographic characteristics, and job satisfaction

Generally speaking, and especially in police work as a male-dominant system, gender can be considered an important determinant of job satisfaction (25). Previous studies on job satisfaction and gender have shown different results. The majority of research indicated that despite worse working conditions (such as gender discrimination, less chance of promotion, and pay differences), women reported higher job satisfaction than their male counterparts even after controlling for a wide range of job characteristics (26, 27). Clark (26) explained this gender-job satisfaction paradox according to women's lower expectations compared to men's rooted in a history of male domination and the lower position of women in the labor market. Accordingly, in younger, higher educated employees, those who work in professional or managerial positions, or those who work in male-dominated workplaces, and thus who have higher expectations about their jobs, the gender gap decreases or disappears. Results of other studies have confirmed this explanation for gender differences in job satisfaction. A study in 14 countries of the European Union (28) indicated that the gender-job satisfaction paradox disappeared in countries with less restriction in access to the labor market for women and with more equal opportunities for them (e.g., the Scandinavian countries). Another study in 32 European countries showed that growing up in contexts with higher gender equality leads to equal expectations between women and men, and the gender gap in job satisfaction is smaller in those countries (29). As Pita and Torregrosa (30) found, the gender gap in job satisfaction has become smaller over this century, and they predict that this paradox will gradually disappear with the advent of more equal working conditions for men and women. In studies of the police field, several studies reported no statistically significant gender differences in Job satisfaction. Dantzker and Kubin (31), in their study among a sample of 2,309 male and 309 female officers in the United States, explained that gender had no significant relationship with job satisfaction. Zhao et al. (25) reported similar results among 199 police officers from a medium-sized police department in the United States. Furthermore, Johnson (32) and Juncaj (4) did not find any significant association between gender and job satisfaction in police officers. Barnett (33) in her study among 930 law enforcement, showed there was no significant difference in overall job satisfaction between male and female officers.

Furthermore, empirical research on determinant factors of job satisfaction in police work studied some other sociodemographic characteristics. Age and job experience (years of service) were studied as two important variables and some of the results indicated a negative relationship between age with a level of job satisfaction in police enforcement (31, 32, 34, 35). Abdulla et al. (36) found a higher job satisfaction among police officers with higher experience, and some other research did not report any relationship between job satisfaction and years of job experience (37, 38). In relation to shifting work, Juncaj (4) reported lower job satisfaction in the police who worked the rotating shift. However, Abdulla et al. (36) did not find any association between shift work and job satisfaction among police officers in their study. Also, police officers who worked at the supervisory/managerial level were more satisfied compared to those who did not have any supervisory/ managerial position (36).

### Stress, gender-based and sexual harassment, and job satisfaction

As mentioned earlier, work-related stress is a predictor of job satisfaction. Therefore, demanding and stressful jobs such as police work can influence feelings about different facets of one's job and might be associated with lower job satisfaction in police officers. According to the transactional theory by Lazarus and Folkman (39) stress is defined as a reciprocal transaction between an individual and their environment and the extent to which a certain environmental event is appraised as threatening and taxing by a person and how capable they are of managing the appraised threat. Work-related stress is an employee's response to work demands and pressures that do not conform to their capabilities, knowledge, resources, and coping skills (40). The consequences of the stressors have been determined by the extent to which the employee appraises the demands threatening and whether they find their resources and capabilities enough to cope with the work demands. Additionally, Cavanaugh et al. (41) explained the challenge-hindrance model (CHM) of stress that classified work stressors into challenge stressors and hindrance stressors categories. According to this model, challenge stressors are the demands that can be stressful but at the same time, are motivating and result in a feeling of accomplishment, development, and satisfaction. On the other hand, hindrance stressors, are the job demands that are taxing and hinder employee development and negatively influence wellbeing and job satisfaction (42). Considering both the transactional theory of stress and the CHM, distinguishing a stressor as a challenge or a hindrance stressor roots in an employee's appraisal of the stressor and the resources for dealing and coping with that.

Police officers deal with operational stress such as being involved in the prevention and investigation of crimes, dealing with criminals and vulnerable people, and being involved with police interventions and the maintenance of public order (43, 44). In addition, as for other jobs, police officers are faced with organizational stress that includes administrative work, supervision, interaction with colleagues, organization support, management, and resources. Alexopoulos et al. (45) in their study among police officers in Greece showed that police officers with higher levels of perceived stress reported lower levels of job satisfaction and lower quality of life. In addition, in a study of officers from police, fire, and ambulance services, Brough (46) found that regarding police officers, organizational stress was a stronger predictor of job satisfaction compared to traumatic experiences and operational stress, while operational demands were predictive of psychological problems and less predictive of job satisfaction.

Work-related gender-based and sexual harassment are considered to be stressors that can increase work-related stress in both genders in any organization (16). According to the definition of the European Parliament and the European Council (47) (Directive 2006/54/EC), sexual harassment is when “any form of unwanted verbal, non-verbal or physical conduct of a sexual nature occurs, with the purpose or effect of violating the dignity of a person, in particular when creating an intimidating, hostile, degrading, humiliating or offensive environment.” Similarly, gender-based harassment is defined as another type of discrimination related to gender, but of no explicitly sexual nature. From a feminist perspective, sexual and gender-based harassment has been discussed as a type of gender discrimination that roots in and perpetuates the traditional and inferior roles of women in work (48). Police work has been established based on masculine norms and expectations, and female police officers do not conform to pre-defined masculine values (15). Therefore, female police officers who challenge the traditional and gendered position, and other officers with non-normative gender and sexual expressions who are not defined as masculine enough, are at higher risk of sexual and gender-based harassment (49, 50).

Sexual and gender-based harassment can lead to negative outcomes for both the individual and the organization, including decreased job satisfaction, higher risk of psychological problems, lower organizational commitment, and higher turnover intentions (51, 52). Hershcovis et al. (53) study of the police organization in the UK showed that sexual harassment from organization insiders negatively affected police officers' job satisfaction, while sexual harassment from outsiders was not associated with job dissatisfaction or other adverse outcomes.

Consistent with the transactional stress theory and the CHM, the current study explores whether the appraised work demands (work stress subscales and sexual and gender-based harassment) by Swedish police officers can influence their job satisfaction.

## Methods

The Swedish police authority in the Stockholm region started a project so-called Mareld to reduce crime and increase safety and security for authorities, businesses, and citizens living in vulnerable areas in the Stockholm region (Botkyrka, Rinkeby, and Södertälje) (54). According to the Swedish police authority (55), particularly vulnerable areas are defined as areas with low socioeconomic status and high crime rates and insecurity. One of the objectives of the Mareld project was to improve the working environment of the police officers working in those areas. In this current cross-sectional study we tried to contribute to the Mareld project by taking into account job satisfaction and gender-based and sexual harassment besides sociodemographic characteristics and work-related stress. All police officers working in these areas during the spring of 2020 were asked to participate in the study. Therefore, 510 questionnaires were distributed among the police employees working in the three vulnerable areas in Stockholm. 275 police employees completed the questionnaires (about 54% response rate). In order to protect respondents' confidentiality, the front page of the questionnaires (containing the personal identification number and personal sociodemographic characteristics) was detached from the completed questionnaire by the respondents and sent in a different sealed envelope to the research team. Some questionnaires were omitted during the process of matching with its front page and with the related Mareld questionnaire (containing the Police Stress Identification Questionnaire). Finally, 189 questionnaires completed by police employees (police officers and civil servants) were retrieved. According to the differences between the nature of work between police officers and civil servants and the low number of civil servants, we decided to focus on the police officers group. After excluding 37 civil servants, questionnaires of 152 police officers (including police patrol officers, investigators, and other internal services officers) were included in the data analysis. According to the COVID-19 situation during the data collection period (2 weeks), the distribution of questionnaires and the data collection were performed by contact persons who were police employees from the three local police districts and were educated about the study and data collection process. The respondents were asked to complete the questionnaires after an explanation of the study's aims and processes by contact persons. Participation in the study was voluntary, and the respondents had the freedom to withdraw from the investigation at any time without any negative consequences.

### Instruments

Data collection was conducted using a set of questionnaires, including sociodemographic questions, the Police Stress Identification Questionnaire (PSIQ), sexual and gender-based harassment questions, and the Job Descriptive Index (JDI).

*The sociodemographic questions* were designed to collect sociodemographic information such as the respondent's gender, age, marital status, job experience, type of work, and a few other sociodemographic variables.

*The PSIQ* is a 42-item self-reported instrument used to measure police work stress on a 9-point Likert scale [ranging from “no stress” (0) to “the most stressful level” (9)] and was developed by Ghazinour et al. (56). The items placed in five subscales include organizational stress (17 items with a Cronbach's alpha of 0.93), operational stress (six items with a Cronbach's alpha of 0.93), impact on significant others (seven items with a Cronbach's alpha of 0.88), self-image (six items with a Cronbach's alpha of 0.78), confrontation with death (four items with a Cronbach's alpha of 0.80), and two single items to measure different types of stressors in police work. In this study, we analyzed the data based on the total scores of the stress subscales (the mean score of the items in each subscale), and the single items were not included.

*The sexual and gender-based harassment questions* were designed by the research team based on the Sexual Experiences Questionnaire (57) and the Sexual Harassment Inventory (58) to collect the respondents' gender-based and sexual harassment experiences in police work. The questionnaire consists of 36 questions about police officers' experiences of sexual (27 questions) and gender-based (9 questions) harassment from public citizens, supervisors, and colleagues over the last 12 months. Each question was scored between “never” (0) to “several times in a week” (5). In this study, in order to avoid complicated item-by-item analysis, respondents' answers to the questions were categorized into two groups based on “had at least one experience” or “had no experience” of gender-based and sexual harassment. Those officers who experienced at least one of the items of gender-based or/and sexual harassment questions were categorized as group 1 (had experience of GH/SH) otherwise they were categorized into group 0 (had no experience of GH/SH). Then gender-based and sexual harassment variables as dichotomous variables were analyzed separately and in relation to other variables.

*The JDI* was originally designed by Smith et al. (24) to measure job satisfaction based on the employee's feelings about their job. In 1989 an initial version of the Job in General Index (JIG) was developed to measure global job satisfaction (59). The JDI questionnaire has been revised several times (60–62). The latest version of the questionnaire contains both the JDI and the JIG and includes 90 items (words) to describe job satisfaction in six subscales (in general and in five specific facets): job in general (18 items with a Cronbach's alpha of 0.85), people on present job (18 items with a Cronbach's alpha of 0.87), work on present job (18 items with a Cronbach's alpha of 0.77), pay (nine items with a Cronbach's alpha of 0.85), opportunities for promotion (nine items with a Cronbach's alpha of 0.91), and supervision (18 items with a Cronbach's alpha of 0.79). Each subscale consists of short lists of words (adjectives or short phrases), and respondents can describe their job using one of three answer options–yes (scoring 3), no (scoring 0), or cannot decide (scoring 1). After reversing the value scores for items with negative wording, the total score of each facet is computed based on the mean score of the item scores.

### Statistical analysis

As the dependent variables (job satisfaction subscales) were normally distributed (tested by Kolmogorov-Smirnov-test), *T*-tests were applied for testing group differences on the continuous variables, and χ2 tests were used for categorical variables. Pearson correlation coefficients were used to indicate associations between continuous variables (job satisfaction subscales, stress subscales, age, and job experience). The variables that had significant associations with the dependent variables in bivariate analysis were applied to multivariate analysis. Hierarchical multiple regression analysis was used to test for the predictive value of sociodemographic variables (job experience, gender, type of work, and managerial position) in the first block, and work stress (organization stress, operational stress, self-image, and confrontation with death) in the second block with job satisfaction domains as dependent variables. The Cronbach's alpha analysis was applied to assess the internal consistency of the job satisfaction questionnaire and the Police Stress Identification Questionnaire. The analysis was performed in SPSS v.26.

## Results

The participants were 30% female and 70% male, and none mentioned any other type of gender. The average age of the participants was 39.9 ± 9.5 years old (range 25–64 years). Most of the respondents (84%) were in a type of intimate relationship (marriage or partnership) and had a university education (81%). Furthermore, 61% of the male officers were working as police patrol officers, whereas the majority of the female police officers (64%) were working in internal work such as investigation, reception, and other types of police work. In total, 32% of the participants were in a managerial position, but the percentage distribution was significantly higher among males (36.4%) compared to females (20%). About 62% of the female officers were working on the day shift, while 62% of the male police officers worked on two or three-shift plans (Table 1).

**Table 1:** Characteristics of the sample by gender.

	**Male (107, 70.4%)**	**Female (45, 29.6%)**	**Total (*****N***=**152)**	***z***	***P*** **-value**
	**Mean** ±**SD or** ***N*** **(%)**	**Mean** ±**SD or** ***N*** **(%)**	**Mean** ±**SD or** ***N*** **(%)**		
Age	39.24 ± 9.41	41.61 ± 9.58	39.92 ± 9.49	1.383	0.169
Job experience	11.95 ± 10.88	14.60 ± 11.11	12.74 ± 10.98	1.360	0.176
Marital status				
Single	14 (13.2%)	10 (22.2%)	24 (15.9%)	χ^2^ =1.920	0.166
Married/co-habitant/living apart together	92 (86.8%)	35 (77.8%)	127 (84.1%)		
Education				
Elementary school & gymnasium	21 (20%)	7 (15.6%)	28 (18.7%)	χ^2^ =0.410	0.522
University	84 (80%)	38 (84.4%)	122 (81.3%)		
Managerial position				
Yes	39 (36.4%)	9 (20%)	48 (31.6%)	χ^2^ =3.967	0.046
No	68 (63.6%)	36 (80%)	104 (68.4%)		
Type of work				
External services	62 (60.8%)	16 (35.6%)	78 (53.1%)	χ^2^ =7.980	0.005
Internal services	40 (39.2%)	29 (64.4%)	69 (46.9%)		
Shift plan				
Day time	41 (38.3%)	28 (62.2%)	69 (45.4%)	χ^2^ =7.302	0.007
Two/three-shift work	66 (61.7%)	17 (37.8%)	83 (54.6%)		

Both male and female police officers reported the highest satisfaction in “people on your present job.” The lowest score of job satisfaction in both male and female police officers was related to “opportunity for promotion” followed by “pay.” Although female police officers reported less satisfaction in “job in general,” “work on present job,” “pay,” and “opportunity for promotion” compared to their male counterparts, there were no significant differences between the subscales of job satisfaction between male and female police officers (Table 2).

**Table 2:** Job satisfaction subscales, work stress subscales, and gender-based and sexual harassment by gender.

	**Male**	**Female**	**Total**	***t***	***P*** **-value**
	**Mean** ±**SD or** ***N*** **(%)**	**Mean** ±**SD or** ***N*** **(%)**	**Mean** ±**SD or** ***N*** **(%)**		
Job satisfaction					
People on your present job	46.13 ± 8.95	47.49 ± 6.61	46.52 ± 8.35	0.900	0.369
Job in general	44.62 ± 8.70	42.53 ± 9.33	44.02 ± 8.90	−1.298	0.196
Work on present job	38.32 ± 7.85	35.40 ± 10.09	37.48 ± 8.62	−1.704	0.093
Pay	33.68 ± 14.52	31.35 ± 15.32	33.01 ± 14.74	–0.876	0.382
Opportunities for promotion	21.66 ± 17.46	18.88 ± 17.25	20.87 ± 17.39	–0.885	0.378
Supervision	42.56 ± 8.82	43.47 ± 8.32	42.82 ± 8.66	0.577	0.565
Work stress					
Organizational stress	2.74 ± 1.51	3.11 ± 1.37	2.85 ± 1.47	1.278	0.201
Impact on significant others	3.56 ± 1.91	4.41 ± 2.32	3.81 ± 2.07	−2.216	0.027
Operational stress	3.27 ± 1.94	4.12 ± 2.36	3.52 ± 2.10	−2.160	0.031
Self-image	2.72 ± 1.49	3.21 ± 1.57	2.87 ± 1.53	−1.791	0.073
Death confrontation	2.70 ± 1.74	3.17 ± 1.80	2.84 ± 1.77	−1.592	0.111
Gender-based harassment	22 (20.6%)	19 (44.2%)	41 (27.3%)	χ^2^=8.620	0.003
Sexual harassment	52 (48.6%)	19 (44.2%)	71 (47.3%)	χ^2^=0.240	0.625

“Impact on significant others” was reported as the highest work-related stress in police officers. Female police officers scored higher in all aspects of work-related stress compared to male officers, although the differences were only statistically significant in “impact on significant others” and “operational stress” (Table 2). In addition, 21% of the male and 44% of the female police officers experienced one to several instances of gender-based harassment (range 1–8 incidents), and the difference was statistically significant. Also, about 49% of the male police officers and 44% of the female police officers reported experiencing sexual harassment, but the difference between the male and female police officers was not statistically significant.

The older and more experienced the officers were, the less satisfaction was reported in “job in general” and more satisfaction was reported in “pay.” Police officers who had managerial positions reported statistically higher job satisfaction in “pay” and “opportunities for promotion” but lower satisfaction with “supervision” compared to other officers. Comparing job satisfaction in the two work type groups showed that police patrol officers (external service) had higher job satisfaction in “job in general” (*t* = 3.365, *P* < 0.001), “work in present job” (*t* = 3.361, *P* = 0.001), “opportunity for promotion” (*t* = 1.992, *P* = 0.048), and “supervision” (*t* = 2.048, *P* = 0.042) compared to those officers who worked in internal services. Also, police officers who worked on a two or three-shift work plan reported greater job satisfaction in “job in general” (*t* = −3.405, *P* = 0.001), “work in present job” (*t* = −3.680, *P* = 0.049), and “supervision” (*t* = −2.167, *P* = 0.032), but less satisfaction in “pay” (*t* = 1.985, *P* = 0.001) than those who worked only a day-shift plan (Table 3).

**Table 3:** Bivariate correlation/association between job satisfaction subscales and socio-demographic variables.

	**People on your present job**	**Job in general**	**Work on present job**	**Pay**	**Opportunities for promotion**	**Supervision**
Age	0.029/0.727	–0.233/0.004	–0.153/0.063	0.309/ <0.001	–0.143/0.083	–0.120/.164
Job experience	0.007/0.929	–0.242/0.003	–0.151/0.067	0.382/ <0.001	–0.156/0.058	–0.158/0.055
Type of work	–0.478/0.634	3.365/ <0.001	3.361/0.001	−1.661/.099	1.992/.048	2.048/.042
External services	46.28 ± 9.75	46.21 ± 8.66	39.72 ± 7.04	31.05 ± 13.03	23.44 ± 17.17	43.99 ± 8.39
Internal services	46.96 ± 6.66	41.31 ± 8.81	34.93 ± 9.68	35.19 ± 16.46	17.79 ± 16.83	41.04 ± 8.89
Managerial position	−1.492/0.138	–0.769/0.443	0.050/0.960	2.356/0.020	3.456/ <0.001	−3.974/ <0.001
Yes	45.02 ± 9.93	43.19 ± 9.85	37.53 ± 7.45	37.15 ± 14.01	27.87 ± 18.83	38.85 ± 10.97
No	47.20 ± 7.47	44.40 ± 8.46	37.46 ± 9.02	31.13 ± 14.74	17.67 ± 15.77	44.63 ± 6.69
Shift plan	0.553/.581	−3.405/ <0.001	−3.680/ <0.049	1.985/0.001	−1.712/0.089	−2.167/.032
Day time	46.94 ± 6.69	41.36 ± 8.78	34.63 ± 9.61	35.70 ± 16.13	18.18 ± 17.28	41.13 ± 8.93
Two/three-shift	46.18 ± 9.50	46.17 ± 8.46	39.78 ± 6.97	30.84 ± 13.22	23.04 ± 17.27	44.18 ± 8.25

Job satisfaction domains and different aspects of work-related stress indicated that job satisfaction in “job in general,” “pay,” and “supervision” were significantly associated with organizational stress. In addition, all work-related stress subscales were negatively correlated with job satisfaction in “pay.” Also, officers with higher stress scores in “operational stress,” “self-image” and “confrontation with death” reported lower satisfaction in “pay,” whereas “operational stress” had a positive relationship with satisfaction in “opportunities of promotion” (Table 4). There were no significant associations between any of the job satisfaction subscales and having experience of gender-based harassment. Bi-variate analysis indicated higher job satisfaction in “work on present job” in the officers with experience of sexual harassment, but this relationship disappeared after controlling for the variable “type of work” in multivariate analysis (*F* = 2.350, *P* = 0.128) (Table 4).

**Table 4:** Bivariate correlation/association between job satisfaction subscales and work stress subscales, gender-based and sexual harassment.

	**People on your present job**	**Job in general**	**Work on present job**	**Pay**	**Opportunities for promotion**	**Supervision**
Work stress						
Organization stress	–0.060/0.468	–0.212/0.009	–0.118/0.152	–0.302/ <0.001	–0.050/0.540	–0.214/0.009
Significant others	0.039/0.633	0.055/0.505	0.074/0.366	–0.1850/0.023	0.082/0.319	0.081/0.326
Operational stress	0.075/0.362	0.015/0.852	0.127/0.121	–0.172/0.036	0.172/0.035	0.045/0.586
Self-image	–0.034/0.683	–0.017/0.836	0.058/0.477	–0.162/0.048	0.107/0.193	0.020/0.803
Death confrontation	–0.031/0.708	–0.143/0.081	0.027/0.742	–0.187/0.022	0.115/0.161	0.052/0.531
Gender-based harassment	0.874/0.383	0.992/0.323	1.461/0.146	0.414/0.680	–0.305/0.761	0.471/0.638
No	46.85 ± 8.68	44.49 ± 8.84	38.15 ± 8.72	33.28 ± 14.87	20.72 ± 17.74	43.01 ± 8.61
Yes	45.50 ± 7.46	42.85 ± 9.16	35.83 ± 8.25	32.15 ± 14.70	21.70 ± 16.58	45.25 ± 9.00
Sexual harassment	1.273/0.205	−1.264/0.208	−2.211/0.029	1.533/0.127	–0.644/0.520	–0.788/0.432
No	47.32 ± 7.54	43.17 ± 8.32	36.05 ± 9.21	34.74 ± 14.87	20.10 ± 17.90	42.27 ± 9.03
Yes	45.58 ± 9.16	45.01 ± 9.52	39.14 ± 7.69	31.04 ± 14.54	21.94 ± 16.89	43.39 ± 8.33

In the hierarchical multiple regression analyses (method: enter), the job satisfaction domains were used as the dependent variables and the sociodemographic variables of gender, type of work, job experience, and managerial position were entered as block 1 and the work-related stress domains were entered as block 2. The following results were obtained (Table 5).

**Table 5:** Hierarchical multiple regressions with work stress (Block 2), as independent and job satisfaction subscales as dependent variables controlling for socio-demographic variables (Block 1) in police officers (method: enter).

**Dependent variable**	**Adjusted** ***R**^2^*	**F**	***P*** **-value**	**Variables with significant standardized Beta (**β, ***t***, ***P*****)**
People on present job				
Model 1	–0.021	0.278	0.892	–
Model 2	–0.011	0.828	0.592	–
Job in general				
Model 1	0.068	3.604	0.008	–
Model 2	0.204	5.033	<0.001	Organizational stress (–0.433, −3.753, <0.001); impact significant others (0.267, 2.278,0.024); death confrontation (–0.335, −3.172, 0.002)
Work on present job				
Model 1	0.058	3.181	0.016	Type of work (–0.238, −2.317,0.022)
Model 2	0.118	3.118	0.002	Organizational stress (–0.370, −3.045,0.003); operational stress (0.257, 2.107,0.037)
Pay				
Model 1	0.168	8.188	<0.001	Job experience (0.439, 4.577, <0.001)
Model 2	0.256	6.428	<0.001	Job experience (0.440, 4.740, <0.001); managerial position (–0.169, −2.248,0.026); organizational stress (–0.486, −4.352, <0.001); self-image (0.356, 2.472,0.015)
Opportunities for promotion				
Model 1	0.086	4.337	0.002	Managerial position (–0.291, −3.528, <0.001)
Model 2	0.129	3.344	0.001	Managerial position (–0.300, −3.689, <0.001); Organizational stress (–0.336, −2.780, 0.006)
Supervision				
Model 1	0.101	4.971	<0.001	Managerial position (0.294, 3.598, <0.001)
Model 2	0.190	4.692	<0.001	Managerial position (0.270, 3.441, <0.001); organizational stress (–0.504, −4.330, <0.001)

*Model 1: The sociodemographic variables (block 1) as independent variables and each job satisfaction subscale as dependent variable.*

*Model 2: The Sociodemographic variables (block 1) combined with the Work stress subscales as independent variables and each job satisfaction subscale as dependent variable*.

The variance in the sociodemographic variables from block 1 could explain between 5.8% of the variance in “work on present job” and 16.8% in “supervision.”

The variance in the sociodemographic variables combined with the stress domains (block 1 and block 2) explained between 11.8% of the variance in “work on present job” and 25.6% in “pay.” Organizational stress was most often of predictive impact related to various job satisfaction domains in police officers, and after controlling for other variables between 33.6% of the variance in “opportunities for promotion” and 50.4% in “supervision” were explained by organizational stress.

## Discussion

Our findings did not show significant differences in job satisfaction between male and female police officers. This result confirms other studies in police officers (31, 63–65) showing only slight role of gender on job satisfaction. However, our finding is in contrast to some studies in other professions than police work showing higher job satisfaction among women compared to men (66–68). The small gender difference in job satisfaction in our results could be explained based on more gender-equal working conditions in Sweden in general and in the Swedish police organization. The studies of Perugini and Valdisavljevic (29) and Pita and Torregrosa (30) indicate that working in an environment with equal working conditions for women and men decreases the gender gap in job satisfaction. Sweden has had a leading position in gender equality among European countries since 2010 (69). Regarding the Swedish police organization, an expressed ambition to promote gender equality and an almost equal gender distribution with 46% female police employees (70) might have contributed to high expectations of gender-equal conditions. However, it should also be noted that only around 30% of the police officers are women and that the increase of female police officers is much less compared to other categories of police employees, such as female civil servants. Other gender equality aspects than gender distribution must also be considered, for example, differences in status between police officers and civil servants, and how masculine norms and expectations impact how female police officers are perceived within the police organization and by the public.

Both male and female police officers reported the lowest scores in job satisfaction in “opportunities for promotion” and “pay” dimensions, which showed a common dissatisfaction in these two subscales. According to the findings of Brunetto and Farr-Wharton (71) and Dantzker and Surrette (72), which demonstrated a negative role of dissatisfaction with pay and promotion in officers' job satisfaction, extrinsic factors such as salary and more opportunities for job promotion need to be considered for improving job satisfaction among police officers. As Bonifacio (73) explains, officers who are dissatisfied with opportunity for promotion and pay feel that their efforts are not appreciated by the police organization and that the system deprives them of their deserved benefits. This feeling of unfairness and deprivation can result in high employee turnover, psychological pressure, low organizational commitment, poor work performance, and low productivity (74).

The findings of comparing job satisfaction between police patrol officers and internal police officers indicated that the police patrol officers had greater job satisfaction in “job in general” and “working in present job” than the internal officers. This finding was in contrast with some studies that demonstrated that police officers with higher levels of work-related stress reported lower job satisfaction. Higher job satisfaction in police patrol officers (who are usually dealing with greater operational stress) can be explained by a feeling of occupational pride as being a “real police officer” (75). Also, as Johnson (32) explained in his study, variety in work tasks has a positive relationship with job satisfaction, and employees with a variety of work duties reported higher job satisfaction. Police patrol officers encounter a remarkable variety of situations and tasks during their work, and therefore higher job satisfaction in this group can be explained based on the greater variety in their everyday work. Police patrol officers reported higher satisfaction in “supervision” and “opportunities for promotion,” and these can positively affect general job satisfaction in this group. According to the CHM of stress, although patrol work, police operation and job variety are considered stressful, on the other hand, these stressors can act as a motivator and lead them to better performance and a feeling of confidence and satisfaction. There is a need for more research in this field to scrutinize job satisfaction in police patrol officers compared to other groups of employees in police organization.

Among the various subscales of police stressors using the PSIQ, organizational stress showed a negative correlation with the three job satisfaction domains of “job in general,” “pay,” and “supervision.” Also, organizational stress was most often of predictive impact related to various job satisfaction domains in police officers. This finding confirmed the results of other studies that demonstrated organizational stress and organizational characteristics are more crucial predictors of job satisfaction in police officers than operational stress and job characteristics (4, 46). Therefore, organization-related stress such as insufficient pay, lack of organizational support and proper supervision, lack of human resources and equipment, and poor administrative practices can negatively influence job satisfaction more than operational stress and traumatic job events related to the police profession. It seems despite operational stressors, organizational constraints and demands are often considered hindrance stressors that negatively influence work outcomes and employees' job satisfaction (76).

According to our findings, there was no significant association between experience of gender-based and sexual harassment and different subscales of job satisfaction in police officers. These findings were not consistent with other studies (52, 53, 77) reporting negative effects of sexual and gender-based harassment on job satisfaction in police officers. The different findings can be due to the smaller sample size of this study compared to the other studies. Another explanation for this result might be the normalization of gender-based and sexual harassment in police work. As dealing with harassment is relatively frequent for police officers (in this study, “sexual jokes and comments” were reported as the most frequent harassment), especially for female police officers (about 47% reported sexual harassment and 27% reported gender-based harassment), they might appraise harassment as a normal part and characteristic of their job, something they have to endure and cope with, or might regard sexual jokes as inevitable parts of the police jargon (78). Future research should explore and assess police officers' perceptions of harassment and its relationship with job satisfaction.

In conclusion, the current study indicated that police officers felt satisfied with their job in “people on your present job” whereas they reported low job satisfaction in “opportunities for promotion” and “pay.” We saw no evidence of a significant gender gap in job satisfaction. Police patrol officers reported higher satisfaction in “job in general,” “working on present job,” “opportunities for promotion,” and “supervision” compared with their counterparts who worked as internal service officers. Among different subscales of work-related stress, organizational stress had the most significant relationship with job satisfaction subscales. There was no significant relationship between sexual and gender-based harassment with job satisfaction.

### Limitations and directions for future studies

There were several limitations to this study. The main limitation was the low response rate. In addition, some participants' data were lost during the matching process of the questionnaires (the questions section and the front page). Consequently, the small number of participants, especially female officers, limited us from analyzing and comparing data based on more specific types of police work (investigation, reception, patrolling). Also, this problem limited us from taking into account the intersectionality of other factors such as country of birth and gender that might play a role in the results and limited our ability to interpret the results due to, for example, the gender segregation in the type of police work and eventual gender differences in sources of harassment (citizens or colleagues/supervisors). Furthermore, the cross-sectional study design restricted the interpretation of the causal relationships between job satisfaction and other factors.

Further studies on gender-based and sexual harassment in relation to job satisfaction in this group can clarify the role of harassment and police officers' perception of harassment in relation to job satisfaction. In addition, more research in other vulnerable areas and comparing vulnerable and invulnerable areas may provide a holistic picture of stress, gender-based and sexual harassment, and job satisfaction in police work.

### Theoretical and practical contributions

In this study, we tried to assess the relationship between work stress and GH and SH (as two crucial -often ignored- stressors) and job satisfaction in the context of police work by combining the transactional theory of stress and CHM. Our study confirmed some other studies' findings such as the small gender gap in job satisfaction. Considering that the appraisal nature of stress and job satisfaction is an important point in the studies on these concepts, it can explain different findings in this research field. Although we did not find a statistical relationship between GH and SH with job satisfaction, there is a need for more research from a gender perspective, for example about how police officers define, comprehend, and deal with harassment, as well as the impact of organizational aspects and police culture. The findings should be considered by police authorities and decision-makers in police organization for improving the work environment and organizational conditions. According to our findings, organizational stress can negatively affect on job satisfaction of police officers. As dealing with operational stressors and exposure to traumatic events (which are inherent aspects of police work) are mostly inevitable, paying attention to organizational sources of stress–which are more controllable–by police organization can improve working conditions and thus improve police employees' job satisfaction. Furthermore, both male and female police officers reported lower job satisfaction in “opportunities for promotion” and “pay” which means the police authorities in those areas should reconsider these two facets of police work, clarify the process of work promotion and pay more attention to providing fair pay to their employees.

## Data availability statement

The raw data supporting the conclusions of this article will be made available by the authors, without undue reservation.

## Ethics statement

This study was approved by the Regional Ethical Review Board at Umeå University (Dnr 2017/516-31). The participants provided their written informed consent to participate in this study.

## Author contributions

AR, MG, MB, and JH contributed to the conception and design of the study. AR performed the statistical analysis and wrote the first draft of the manuscript. MG, MB, and JH reviewed and revised the manuscript. All authors contributed to manuscript revision, read, and approved the submitted version.

## Funding

This study was funded by The Police Education Unit and Umeå Center for Gender Studies at Umeå University.

## Conflict of interest

The authors declare that the research was conducted in the absence of any commercial or financial relationships that could be construed as a potential conflict of interest.

## Publisher's note

All claims expressed in this article are solely those of the authors and do not necessarily represent those of their affiliated organizations, or those of the publisher, the editors and the reviewers. Any product that may be evaluated in this article, or claim that may be made by its manufacturer, is not guaranteed or endorsed by the publisher.
